# Multi–b-value diffusion-weighted imaging–derived parameters for differentiating high-grade serous ovarian carcinoma from other epithelial ovarian cancers

**DOI:** 10.1007/s11604-025-01813-6

**Published:** 2025-07-01

**Authors:** Tsukasa Saida, Miki Yoshida, Taishi Amano, Masashi Shindo, Reo Nemoto, Takeo Iizuka, Ayumi Shikama, Toyomi Satoh, Takahito Nakajima

**Affiliations:** 1 Department of Radiology, Institute of Medicine, University of Tsukuba, 1-1-1 Tennodai, Tsukuba, Ibaraki 305-8575 Japan; 2 Department of Diagnostic and Interventional Radiology, University of Tsukuba Hospital, 2-1-1 Amakubo, Tsukuba, Ibaraki 305-8576 Japan; 3 Department of Radiology, University of Tsukuba Hospital, 2-1-1 Amakubo, Tsukuba, Ibaraki 305-8576 Japan; 4 Department of Obstetrics and Gynecology, Institute of Medicine, University of Tsukuba, 1-1-1 Tennodai, Tsukuba, Ibaraki 305-8575 Japan

**Keywords:** Ovary, Multiple b, Intravoxel incoherent motion, Diffusion kurtosis imaging, IVIM, DKI

## Abstract

**Objective:**

High-grade serous carcinoma (HGSC) is the most common ovarian cancer subtype, and its differentiation from others is crucial for treatment. This study aimed to evaluate parameters derived from multi–b-value diffusion-weighted imaging (DWI), including apparent diffusion coefficient (ADC), and metrics based on intravoxel incoherent motion (IVIM) and diffusion kurtosis imaging (DKI), for differentiating HGSC from other ovarian cancers.

**Methods:**

We retrospectively analysed patients with primary epithelial ovarian cancer who underwent preoperative MRI including multi-b-value DWI. From the solid tissues of the tumours, diffusion parameters were derived from the multi–b-value DWI data using different models: ADC using a mono-exponential model; the true diffusion coefficient (Di), pseudo-diffusion coefficient (D*), and perfusion fraction (f) using the IVIM model; and kurtosis (K) using the DKI model.

**Results:**

This study included 56 patients with different histological cancer subtypes (mean age, 60 years; range, 24–87 years). The mean values of HGSC compared to the other cancers showed lower ADC (0.58 ± 0.21 × 10⁻^3^ mm^2^/s vs. 0.76 ± 0.18 × 10⁻^3^ mm^2^/s, *p* < 0.001), lower Di (0.37 ± 0.09 × 10⁻^3^ mm^2^/s vs. 0.42 ± 0.15 × 10⁻^3^ mm^2^/s, *p* = 0.201), and lower f (35.79 ± 11.48% vs. 48.01 ± 17.21%, *p* = 0.003), with a higher K (1.06 ± 0.25 vs. 0.84 ± 0.20, *p* = 0.341). Among these parameters, ADC showed the highest diagnostic performance in differentiating HGSC from others, with an area under the receiver operating characteristic curve of 0.79. These trends were particularly pronounced between HGSC and clear cell carcinoma, with significant differences in all parameters except D*. Additionally, K _Mean_ was the only parameter that showed a significant difference between HGSC and endometrioid carcinoma.

**Conclusion:**

Multi–b-value DWI–derived parameters, particularly ADC, may aid in the non-invasive preoperative differentiation of HGSC from other ovarian cancers.

**Secondary Abstract:**

Multi–b-value DWI–derived parameters, especially ADC, demonstrated utility in differentiating high-grade serous carcinoma (HGSC) from other ovarian cancers, highlighting their potential in non-invasive preoperative tumor characterization.

## Introduction

High-grade serous carcinoma (HGSC) is the most common subtype of ovarian cancer and accounts for the majority of deaths due to gynaecological malignancies. Most cases are diagnosed at advanced stages, with the median 5-year survival rate ranging from 15 to 55% depending on the stage and extent of tumour debulking [1]. HGSC and endometrioid carcinoma (EC) typically respond well to platinum-based chemotherapy, whereas clear cell carcinoma (CCC), mucinous carcinoma (MC), and low-grade serous carcinoma (LGSC) are generally more resistant. Moreover, homologous recombination-deficient (HRD)—a key predictor of response to poly ADP-ribose polymerase inhibitors (PARPi)—tends to be more prevalent in HGSC (27–69%)[2, 3] than in CCC (2.3–26%)[2, 4] or MC (0–29%)[2, 5], though there is substantial overlap with EC (24–38%)[2]. Given the similarities in and chemosensitivity and HRD prevalence between HGSC and EC, the clinical value of differentiating these two subtypes may be limited. Therefore, histological subtyping—particularly distinguishing HGSC from other subtypes, especially non-EC types such as CCC and MC—plays a critical role in guiding treatment decisions, as it reflects substantial differences in chemosensitivity and HRD prevalence. Currently, histopathological diagnosis relies on surgical specimens or biopsies, invasive procedures that may not always be feasible due to the patient’s condition or tumour location. Non-invasive imaging techniques, especially MRI, play an important role in characterising ovarian tumours and predicting histological subtypes [6–9]. However, conventional MRI sometimes shows overlapping imaging features, making it difficult to differentiate HGSC from other epithelial ovarian cancer subtypes.

Parameters derived from intravoxel incoherent motion (IVIM) [10] and diffusion kurtosis imaging (DKI) [11], both calculated from multi–b-value diffusion-weighted imaging (DWI), offer complementary information: IVIM provides estimates of perfusion-related and true molecular diffusion, while DKI characterizes microstructural heterogeneity and complexity. These additional biomarkers may better reflect the tumour microenvironment and tissue architecture, potentially aiding in the non-invasive differentiation of HGSC from other subtypes. However, to date, no studies have simultaneously evaluated IVIM and DKI parameters in ovarian cancer. 

Given the clinical importance of accurate histological diagnosis and the limitations of current diagnostic modalities, this study aimed to investigate the utility of multi–b-value DWI–derived parameters, in distinguishing HGSC from other epithelial ovarian cancers, which could lay the groundwork for a more precise preoperative diagnosis and personalised treatment strategies for ovarian cancer.

## Materials and methods

### Patients

Our Institutional Review Board (approval number: R06-203) approved the protocol for this retrospective study, waiving the requirement for written informed consent because of the retrospective nature of the study.

**Fig. 1:**
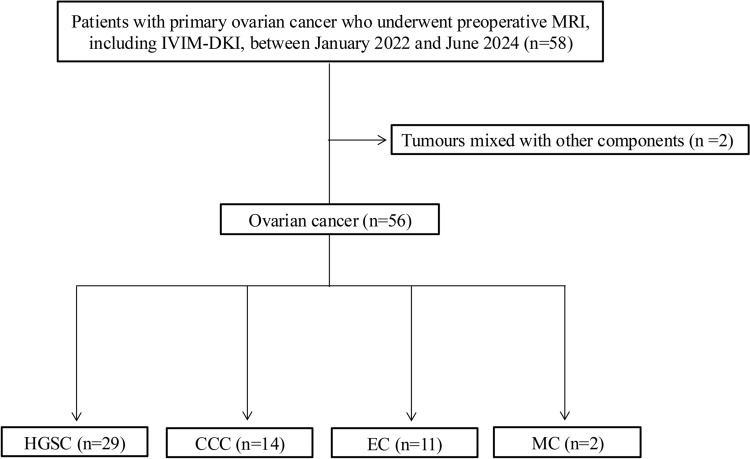
A flowchart for the patient selection process. *CCC* clear cell carcinoma, *DKI* diffusion kurtosis imaging, *EC* endometrioid carcinoma, *HGSC* high-grade serous carcinoma, *IVIM* intravoxel incoherent motion, *MC* mucinous carcinoma

The inclusion criteria were as follows: a) 58 consecutive patients with primary ovarian cancer who underwent preoperative MRI, including multi-b-value DWI, between January 2022 and June 2024, and b) histological subtypes confirmed via surgical removal and pathological examination. The patients were selected from a Radiology Information System. The exclusion criteria were as follows: c) presence of mixed histological types, resulting in the exclusion of two cases. A flowchart of the patient selection process is shown in Fig. 1.

### MRI acquisition

MRIs were acquired using 3 T scanners (Ingenia®, Philips Medical Systems, Amsterdam, Netherlands), and hyoscine butylbromide (20 mg; Buscopan®; Sanofi, France) was injected intramuscularly to all patients immediately prior to the examination, to reduce motion artefacts caused by bowel peristalsis. MRI protocol included T2-weighted imaging (T2WI), T1-weighted imaging (T1WI), and contrast-enhanced fat-saturated T1WI (CE-T1WI) using 5 mmol gadopentetate dimeglumine (Gadovist® 1.0 M; Bayer, Wuppertal, Germany) diluted with saline. DWI with six b-values (0, 50, 100, 1000, 1500, and 2000s/mm^2^) were acquired using a free-breathing single-shot echo-planar imaging sequence to enable subsequent IVIM and DKI model fitting. The imaging parameters were as follows: TR/TE = 5000/80 ms; flip angle = 90°; slice thickness = 4.0 mm; interslice gap = 0 mm; field of view = 28 × 28 cm; matrix size = 144 × 144; parallel imaging factor = 2; number of signal averages = 1. The total acquisition time was 5 min and 45 s.

### Clinical and pathological findings

Clinicopathological findings, including age and histological diagnosis, were obtained from the hospital’s electronic medical records. Histological diagnosis was based on the World Health Organization (WHO) 2020 classification, and borderline tumours were excluded.

### Multi–b-value DWI–derived parameters

The images in the present study were reviewed by three radiologists with 16, 7, and 4 years of post-certification experience specialising in pelvic MRI. They were blinded to each patient’s clinical and pathological findings and independently reviewed the images using the Intellispace Portal V12.1.5 (Philips Medical Systems, Amsterdam, Netherlands).

The region of interest (ROI) was manually set on the solid tissue following the Ovarian Adnexal Reporting Data System MRI [12], with priority given to areas showing high signal on DWI and the lowest values on the apparent diffusion coefficient (ADC) map, excluding haemorrhagic or necrotic regions based on T2WI and CE-T1WI. Relatively small ROIs were set according to a previous statement on uterine sarcomas [13]. The ROIs on the ADC map were transferred to the IVIM and DKI parametric maps. ADC was calculated using the mono-exponential model. From the IVIM model, we derived the true diffusion coefficient (Di), the pseudo-diffusion coefficient (D*), and the perfusion fraction (f). Kurtosis (K) was obtained from the DKI model.

The ADC is calculated using the following equation: S(b)/S_0_ = e^−b⋅ADC^; using 6 b-values of 0, 50, 100, 1000, 1500 and 2,000 s/mm^2^.

The IVIM model is based on a biexponential function and is calculated using the following equation: S(b)/S_0_ = f⋅e^−b・D*^ + (1-f) ⋅e^−b・Di^ [10], where Di represents the pure diffusion of water molecules. In contrast, D* and f are associated with microcapillary perfusion effects. These perfusion effects may attenuate the signal at low b values (b < 200 s/mm^2^). However, at high b-values, the contribution of perfusion-related effects became negligible, allowing the true diffusion component to be detected.

Similarly, the DKI model is derived from a bi-exponential diffusion framework and is expressed as follows: ln (S(b)/S_0_) = − b ⋅ D_k_ + b^2^⋅D_k_^2^⋅K/6 [11], where D_k_ represents diffusion coefficient derived from the kurtosis model (not used as an outcome parameter in this study).

To ensure the reliability of diffusion parameter estimation, signal to noise ratio (SNR) was evaluated for each b-value image. SNR was calculated using a standard method (mean signal in ROI / standard deviation (SD) of background noise).

### Statistical analysis

The means and SDs were calculated for all quantitative data, including age and multi–b-value DWI–derived parameters. These quantitative parameters were compared to differentiate HGSC from the others using the Mann–Whitney U test, and each histological subtype was assessed using the Kruskal–Wallis test, with multiple comparisons corrected using the Dunn–Bonferroni method.

Spearman’s correlation coefficient was used to determine the correlation between the parameters. Independent risk factors were identified using binary logistic regression to construct a risk-prediction model for parameters that could significantly distinguish HGSC from other cancers. Model discrimination and calibration were evaluated using the Hosmer–Lemeshow test.

Additionally, in the receiver operating characteristic (ROC) curve analysis, the area under the receiver operating characteristic curve (AUC) and cutoff values were determined for parameters that could significantly distinguish HGSC from other cancers.

The intraclass correlation coefficient (ICC) was used to assess interobserver reliability.

All statistical analyses were performed using SPSS software (SPSS Statistics 29.0; IBM, New York, NY, USA), and statistical significance was set at *p* < 0.05.

## Results

The present study included 56 patients with various histological cancer subtypes (mean age, 60 years; range, 24–87 years). Table 1 presents patient characteristics.

**Table 1:** Patient characteristics

Pathology	Number of cases	Mean age	Standard deviation of age	Age range
High-grade serous carcinoma	29	66	11	47–87
Clear cell carcinoma	14	53	13	34–72
Endometrioid carcinoma	11	56	18	30–79
Mucinous carcinoma	2	35	11	24–45

**Fig. 2:**
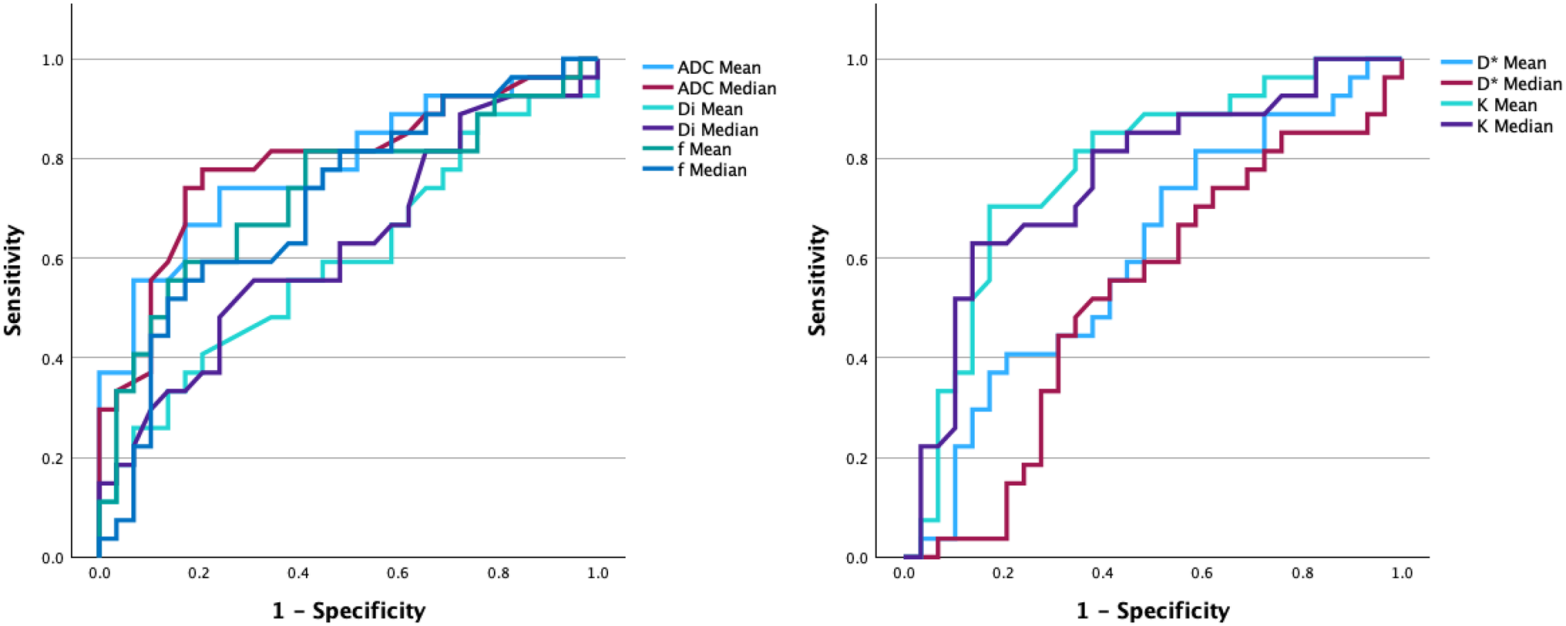
Area under the curve comparison of multi–b-value diffusion-weighted imaging–derived parameters for differentiating high-grade serous carcinoma from other ovarian cancer subtypes

Table 2 presents the multi–b-value DWI–derived parameters, for HGSC and other cancers. The following parameters significantly differed between HGSC and other cancers: ADC _Mean_, ADC _Median_, f _Mean_, f _Median_, and K _Median_. These significant variables were subsequently included as independent variables in logistic regression analysis. The included variables showed a correlation coefficient of r < 0.80, indicating a lack of multicollinearity. Multivariate analysis identified age and ADC _Mean_ as statistically significant predictors (*p* < 0.05) for distinguishing HGSC from other ovarian cancers. The model’s goodness-of-fit was assessed using the Hosmer–Lemeshow test, confirming adequate calibration. The final model achieved an overall predictive accuracy of 82.1%. For the ADC _Mean_, the cut-off was 0.64 with an AUC of 0.79; for the ADC _Median_, the cut-off was 0.65, with an AUC of 0.79. For f _Mean_, the cut-off was 44.68 with an AUC of 0.73; for f _Median_, the cut-off was 44.00 with an AUC of 0.71. For K _Median_, the cut-off was 0.89, with an AUC of 0.71. In Fig. 2, a comparison of the AUCs of the multi–b-value DWI–derived parameters are shown.

**Table 2:** Multi–b-value diffusion-weighted imaging–derived parameters for high-grade serous carcinoma and other ovarian cancers

Parameter		HGSC (n = 29)	The others (n = 27)	*p*	cut-off	AUC	Sensitivity	Specificity
DWI	ADC (10^−3^ mm^2^/s)							
	Mean	0.58 (0.12)	0.76 (0.18)	< 0.001*	0.64	0.79	0.76	0.74
	Median	0.58 (0.13)	0.77 (0.18)	< 0.001*	0.65	0.79	0.79	0.78
IVIM	Di (10^−3^ mm^2^/s)							
	Mean	0.37 (0.09)	0.42 (0.15)	0.201		0.60		
	Median	0.19 (0.11)	0.25 (0.15)	0.108		0.63		
	D* (10^−3^ mm^2^/s)							
	Mean	7.05 (4.12)	5.57 (3.30)	0.176		0.61		
	Median	5.67 (4.02)	5.36 (3.79)	0.964		0.53		
	f (%)							
	Mean	35.79 (11.48)	48.01 (17.21)	0.003*	44.68	0.73	0.83	0.59
	Median	35.27 (16.70)	47.68 (17.00)	0.007*	44.00	0.71	0.79	0.59
DKI	K							
	Mean	1.06 (0.25)	0.84 (0.20)	0.341		0.57		
	Median	1.08 (0.23)	0.87 (0.19)	0.016*	0.89	0.71	0.82	0.60

Note: Data in parentheses indicate standard deviation. *AUC* area under the curve, *CCC* clear cell carcinoma, *DKI* diffusion kurtosis imaging, *DWI* diffusion-weighted imaging, *EC* endometrioid carcinoma,*HGSC* high-grade serous carcinoma, *IVIM* intravoxel incoherent motion, *MC*, mucinous carcinoma. *p < 0.05

**Fig. 3:**
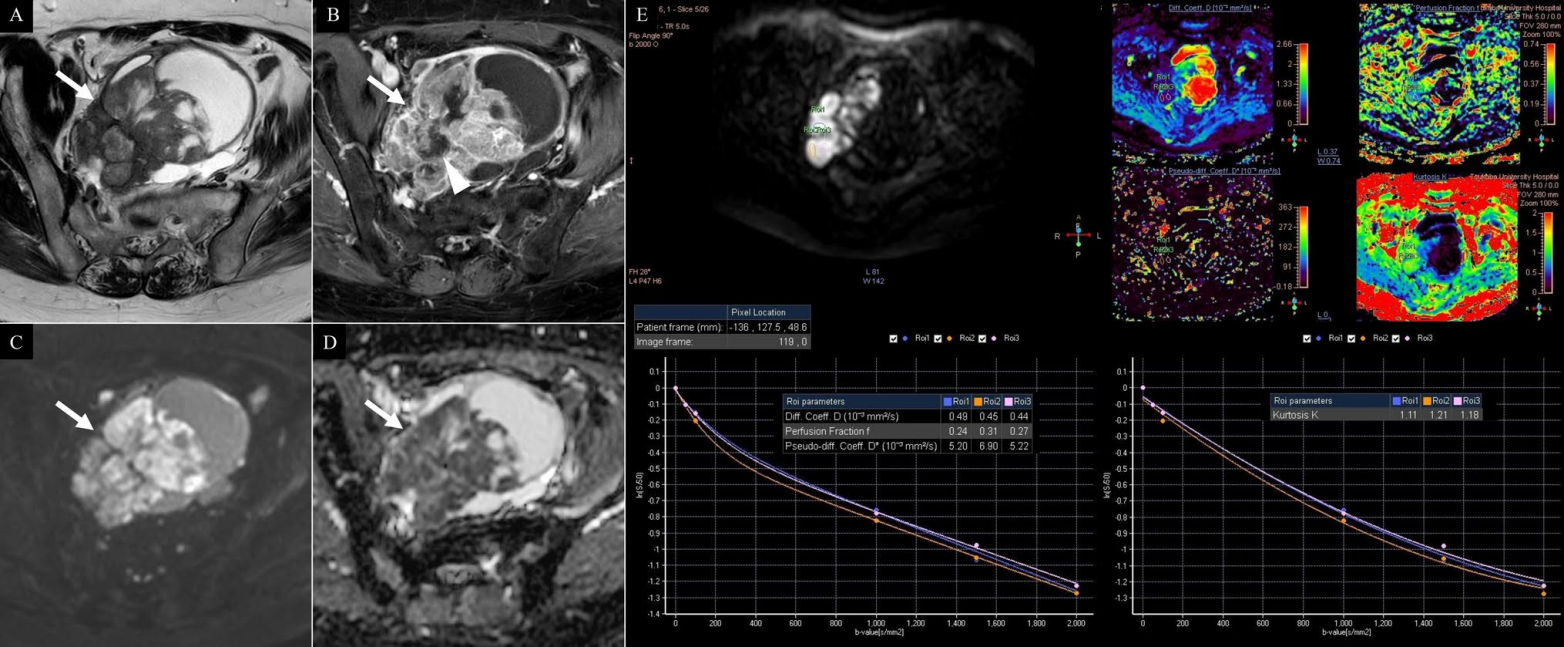
A 67-year-old woman with high-grade serous carcinoma. **A** T2-weighted imaging, **B** contrast-enhanced T1-weighted imaging, **C** conventional diffusion weighted imaging, **D** apparent diffusion coefficient map, **E** intravoxel incoherent motion and diffusion kurtosis imaging analysis (top left: ROI, top right: Di, D*, f, K maps, bottom right: intravoxel incoherent motion model plot, bottom left: kurtosis model plot). A solid and cystic tumour infiltrating the myometrium is located posterior to the uterus (arrows). Post-contrast imaging reveals a central non-enhancing area, indicative of necrosis (**B**: arrowheads). The solid tissue exhibits marked diffusion restriction (**C**, **D** arrows). The mean diffusion-weighted, intravoxel incoherent motion, and diffusion kurtosis imaging parameters measured by three radiologists were as follows: ADC = 0.50 × 10⁻^3^ mm^2^/s, Di = 0.46 × 10⁻^3^ mm^2^/s, D* = 5.77 × 10⁻^3^ mm.^2^/s, f = 27%, and k = 1.17

**Fig. 4:**
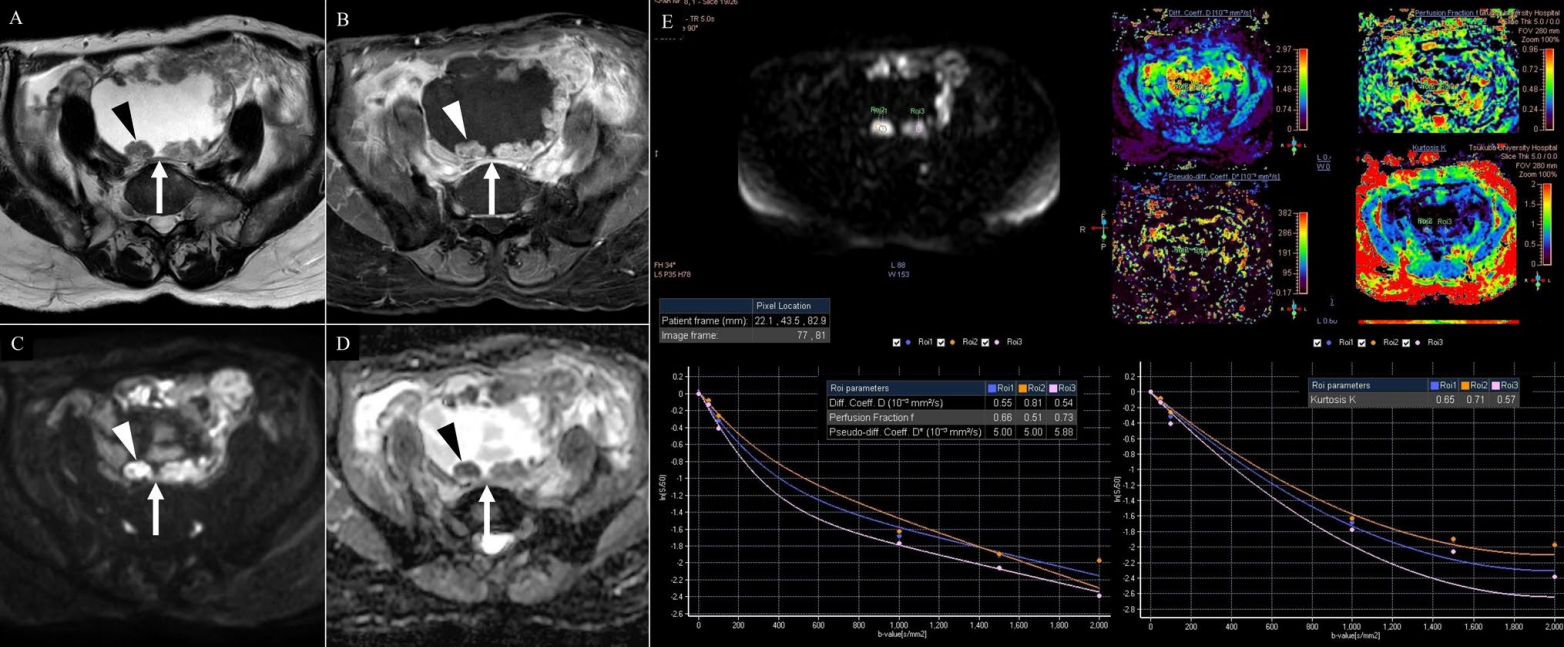
A 72-year-old woman with clear cell carcinoma. **A** T2-weighted imaging, **B** contrast-enhanced T1-weighted imaging, **C** conventional diffusion weighted imaging, **D** apparent diffusion coefficient map, **E** intravoxel incoherent motion and diffusion kurtosis imaging analysis (top left: ROI, top right: Di, D*, f, K maps, bottom right: intravoxel incoherent motion model plot, bottom left: kurtosis model plot). A unilocular cyst is located just beneath the anterior abdominal wall (arrows), containing multiple mural nodules (**A** arrowhead) that exhibit low signal intensity on T2-weighted imaging. Post-contrast imaging shows relatively strong enhancement of the mural nodules (**B** arrowhead), which also correspond to areas of marked diffusion restriction (**D**, **E**: arrowheads). The mean diffusion-weighted, intravoxel incoherent motion, and diffusion kurtosis imaging parameters measured by three radiologists were as follows: ADC = 0.69 × 10⁻^3^ mm^2^/s, Di = 0.63 × 10⁻^3^ mm^2^/s, D* = 5.29 × 10⁻^3^ mm.^2^/s, f = 63%, and k = 0.63

**Fig. 5:**
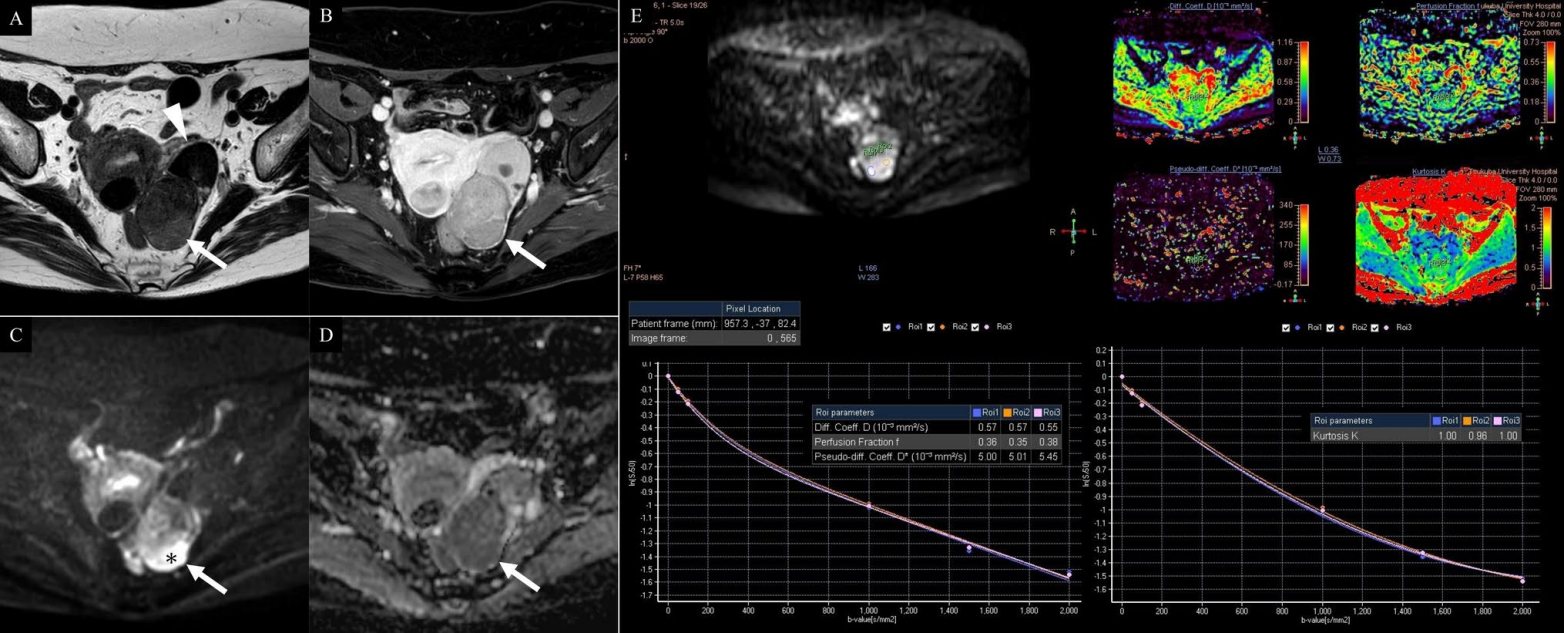
A 72-year-old woman with endometrioid carcinoma. **A** T2-weighted imaging, **B** contrast-enhanced T1-weighted imaging, **C** conventional diffusion weighted imaging, **D** apparent diffusion coefficient map, **E** intravoxel incoherent motion and diffusion kurtosis imaging analysis (top left: ROI, top right: Di, D*, f, K maps, bottom right: intravoxel incoherent motion model plot, bottom left: kurtosis model plot). A solid tumour is present in the left adnexa (arrows), accompanied by a component showing markedly low signal intensity on T2-weighted imaging (**A** arrowhead), which is suspected to be an endometriotic cyst. The findings suggest ovarian carcinoma arising from an endometriotic cyst. The solid tissue demonstrates homogeneous enhancement after contrast administration (**B** arrow), with diffusion restriction that is heterogeneous (**C**, **D** arrows). Strong diffusion restriction is observed along the posterior margin of the solid tissue (asterisk). The mean diffusion-weighted, intravoxel incoherent motion, and diffusion kurtosis imaging parameters measured by three radiologists were as follows: ADC = 0.70 × 10⁻^3^ mm^2^/s, Di = 0.56 × 10⁻^3^ mm^2^/s, D* = 5.15 × 10⁻^3^ mm.^2^/s, f = 36%, and k = 0.99

Table 3 shows the multi–b-value DWI–derived parameters for each histological subtype. In comparing histological subtypes, HGSC showed significantly lower ADC _Mean,_ ADC _Median_, Di _Mean_, Di _Median_, f _Mean_, and f _Median_ and significantly higher K _Mean_ and K _Median_ than CCC. Additionally, HGSC had a significantly higher K _Mean_ than EC, whereas EC had a significantly lower ADC _Mean_ and ADC _Median_ than CCC. Figures 3, 4, and 5 show MR images and IVIM-DKI analysis results for HGSC, CCC, and EC, respectively.

**Table 3:** Multi–b-value diffusion-weighted imaging–derived parameters for each histological subtype

Parameter		HGSC (n = 29)	CCC (n = 14)	EC (n = 11)	MC (n = 2)	*p*
DWI	ADC (10^−3^ mm^2^/s)					
	Mean	0.58 (0.12)	0.87 (0.14)	0.65 (0.13)	0.65 (0.24)	< 0.001* for HGSC vs. CCC, 0.007 for EC vs. CCC
	Median	0.58 (0.13)	0.88 (0.14)	0.67 (0.13)	0.61 (0.22)	< 0.001* for HGSC vs. CCC, 0.011 for EC vs. CCC
IVIM	Di (10^−3^ mm^2^/s)					
	Mean	0.37 (0.09)	0.47 (0.17)	0.36 (0.10)	0.36 (0.05)	0.031* for HGSC vs. CCC
	Median	0.37 (0.11)	0.47 (0.19)	0.38 (0.10)	0.40 (0.11)	0.040* for HGSC vs. CCC
	D* (10^−3^ mm^2^/s)					
	Mean	7.05 (4.12)	6.01 (3.65)	5.06 (2.84)	5.29 (2.54)	
	Median	5.67 (4.02)	7.80 (8.97)	4.68 (2.78)	5.79 (2.72)	
	f (%)					
	Mean	35.79 (11.48)	51.72 (16.94)	44.68 (15.67)	40.27 (20.57)	0.002* for HGSC vs. CCC
	Median	35.27 (16.70)	51.84 (16.25)	43.98 (15.28)	38.95 (22.35)	0.002* for HGSC vs. CCC
DKI	K					
	Mean	1.06 (0.25)	0.78 (0.18)	0.89 (0.19)	1.03 (0.19)	< 0.001* for HGSC vs. CCC, 0.045* for HGSC vs. EC
	Median	1.08 (0.23)	0.81 (0.17)	0.91 (0.20)	1.02 (0.20)	< 0.001* for HGSC vs. CCC

Note: Data in parentheses indicate standard deviation. *CCC* clear cell carcinoma, *DKI* diffusion kurtosis imaging, *DWI* diffusion-weighted imaging, *EC* endometrioid carcinoma, *HGSC* high-grade serous carcinoma, *IVIM* intravoxel incoherent motion, *MC* mucinous carcinoma. *p < 0.05

The average ROI size was 38 mm^2^ (SD, 27 mm^2^), with a minimum of 9 mm^2^. In all cases, the mean SNR exceeded 9 across all b-values, ensuring reliable parameter fitting.

ICC values for various diffusion parameters were as follows: ICC (2,1) for ADC was 0.92, ICC (2,1) for Di was 0.69, ICC (2,1) for D* was 0.66, ICC (2,1) for f was 0.70, and ICC (2,1) for K was 0.91.

## Discussion

Overall, HGSC tended to have lower ADC, Di, and f values and higher K values than other ovarian cancers. Significant differences were observed in the ADC _Mean_, ADC _Median_, f _Mean_, f _Median_, and K _Median_, with the ADC _Mean_ showing the highest AUC. In the comparison between the histological subtypes, significant differences were observed in all parameters, except for D* between HGSC and CCC, and K_Mean_ was the only parameter that showed a significant difference between HGSC and EC.

Multi–b-value DWI enables more detailed analysis of tissue microstructure by allowing separation of multiple diffusion-related parameters. ADC reflects both diffusion and perfusion and is influenced by cellularity, tortuosity of the extracellular/extravascular space, and cell membrane density, based on differences in water proton mobility within tissues [14]. The multi-point method offers more reliable and consistent ADC measurements with less variability compared to the two-point method [15]. Di represents the true diffusion coefficient of water molecules in tissue after excluding microcirculation perfusion. This parameter reflects the intrinsic mobility of water molecules and is influenced by cell density, extracellular space curvature, cell membrane integrity, and liquid viscosity. The Di value decreases when tissues contain more cells, have reduced intercellular space, and have a higher nucleus-to-cytoplasm ratio. As a result, malignant tumours consistently exhibit lower Di values than benign tumours or normal tissues [16–21]. On the other hand, D* is considered to be associated with blood flow in the microvasculature [22–24]. It has been reported to be positively proportional to the average blood flow velocity and capillary segment length, and higher D* values are thought to reflect increased microcirculatory perfusion within the tissue [25, 26]. Similar to D*, f is regarded as a perfusion-related parameter. The f represents the fraction of water molecules moving with microcirculation in capillaries and small vessels. It is believed to reflect the level of blood perfusion related to microvessel density and vascular lumen size. A higher f is generally interpreted as indicating more active blood flow, while a lower f suggests reduced or restricted microcirculation [25, 27]. The DKI model incorporates K, a parameter that quantifies deviations from the Gaussian behaviour caused by tissue heterogeneity, which is generally proportional to the heterogeneity and complexity of the tissue microstructure [11].

Only a few studies have investigated the applications of IVIM and DKI in ovarian cancer. Song et al. used IVIM to differentiate borderline from malignant tumours and found that ADC and Di were higher, while f was lower in borderline tumours. These parameters also correlated with Ki-67 expression and microvessel density [28]. Wang et al. compared type 1 and type 2 ovarian cancers, showing significantly lower ADC, Di, and f values in type 2 cancers, though no difference was observed in D* [29]. Le et al. reported that CCC and EC tended to show higher ADC and Dk values and lower K values compared to HGSC, but did not perform formal statistical comparisons or evaluate IVIM parameters [30]. The trend observed in these studies, where HGSC exhibited lower ADC, Di, and f values but higher K values than other cancers, is consistent with the current study’s findings. The numerical differences may result from variations in models, differences in ROI selection, and, additionally, the larger number of cases included in our study compared to previous reports. Unlike earlier methods, we identified areas with low ADC values and evaluated them using small ROIs, as recommended for uterine sarcoma [13]. Ovarian cancer is a heterogeneous tumour; however, its pathological diagnosis is based on the most malignant region. Therefore, focusing on areas that appear most malignant is logical and straightforward. This approach is practical and well-suited for clinical applications.

ADC reflects both diffusion and perfusion and is sensitive to tumour heterogeneity, which may explain its superior diagnostic performance in distinguishing between HGSC and other cancers, including HGSC versus CCC and CCC versus EC. In contrast, Di reflects only pure diffusion and excludes perfusion-related variability, making it less affected by heterogeneity within the ROI. This stabilizes the Di values but likely led to the absence of significant differences between HGSC and other cancers. However, in subtype comparisons, a significant difference in Di values were observed between HGSC and CCC, and another possible explanation is that this difference may have been offset when other cancer subtypes were included in the CCC group. Among the IVIM parameters, the perfusion-related metrics (D* and f) showed lower reproducibility [16, 29, 31], consistent with prior studies; D* exhibited the lowest ICC in our data. Although Song et al. found that malignant tumours had higher f values than borderline tumours [28], our study, consistent with previous findings, demonstrated lower f values in HGSC, a more aggressive subtype. This may be attributed to necrosis and hypoxia leading to the destruction of microvascular structures. K values were elevated in HGSC, even within the most malignant regions, suggesting marked intratumoural heterogeneity. A significant difference in K _Median_ but not K _Mean_ between HGSC and other cancers implies high variability between ROIs, possibly reflecting microscale structural complexity as reported by Maiuro et al. [32]. HGSC is characterized by high cellularity, nuclear atypia, and complex papillary structures, whereas CCC consists of relatively uniform clear cells with less architectural complexity [33], and EC typically shows glandular differentiation and lower cellular density [34]. These histological differences—and additionally, differences in cellular proliferation as reflected by Ki-67 expression, which is generally higher in HGSC than in CCC and EC [35, 36], —may underlie the elevated K values observed in HGSC. Furthermore, Deen et al. reported that K is a potential biomarker for predicting response to neoadjuvant chemotherapy in HGSC, with higher K values linked to better outcomes [37].

This study had several limitations. First, it was retrospective and limited to cases captured using a single MRI system, which may have introduced selection bias. Second, the number of MC cases was small, and rare epithelial malignancies such as malignant Brenner tumours were not included. Third, pathological validation, such as microvessel density analysis, was not performed. Forth, due to the limited number of low b-values, the derived D* and f values may not fully reflect true perfusion but instead reflect fast and slow diffusive compartments. Therefore, the results should be interpreted as a bi-exponential approximation rather than strict IVIM perfusion parameters. Fifth, the ROI measurement method differs from previous reports; however, our measurement method is straightforward and clinically practical. Finally, future studies with larger and more diverse cohorts are needed to validate the generalizability of our findings.

In conclusion, HGSC was characterised by lower ADC and f values and higher K values than other ovarian cancer types. Among all parameters, ADC derived from multi–b-value DWI demonstrated the highest diagnostic performance in distinguishing HGSC from other ovarian cancers, surpassing the other multi–b-value DWI derived parameters. In comparisons with individual histological subtypes, HGSC exhibited significantly lower ADC, Di, and f values and higher K values than CCC, and only K _Mean_ was useful in distinguishing HGSC from EC.

## Data Availability

The datasets used and analysed during the current study are available from the corresponding author on reasonable request.
